# Closing the Gaps: An Integrative Review of Yoga’s Benefits for Lymphedema in Breast Cancer Survivors

**DOI:** 10.3390/life14080999

**Published:** 2024-08-11

**Authors:** Sara Freguia, Daniela Platano, Danilo Donati, Federica Giorgi, Roberto Tedeschi

**Affiliations:** 1 Department of Biomedical and Neuromotor Sciences, Alma Mater Studiorum, University of Bologna, 40136 Bologna, Italy; 2 Physical Medicine and Rehabilitation Unit, IRCCS Istituto Ortopedico Rizzoli, 40136 Bologna, Italy; 3 Physical Therapy and Rehabilitation Unit, Policlinico di Modena, 41125 Modena, Italy; 4 Clinical and Experimental Medicine PhD Program, University of Modena and Reggio Emilia, 41124 Modena, Italy; 5 Pediatric Physical Medicine and Rehabilitation Unit, IRCCS Institute of Neurological Sciences of Bologna, Via Altura 3, 40124 Bologna, Italy

**Keywords:** lymphedema, yoga, breast cancer, rehabilitation

## Abstract

Background: Dissection of the axillary lymph nodes during surgery for breast cancer with lymph node involvement is burdened by a complication: lymphedema. Approximately half of women undergoing axillary dissection suffer from it, with a notable impact in terms of perceived discomfort, presented quality of life, and alteration of body image. There is also no shortage of problems in the patient’s social and professional life. Methods: The present review aims to select Randomized Controlled Trials (RCTs) present in the literature regarding the effects of yoga as an alternative therapy in patients with breast cancer-related lymphedema. A search of four databases was undertaken: Cochrane, Pubmed, Scopus, and Web of Science. The searches were conducted on 19 May 2024, and updated to 30 June 2024 without date limits. RCTs without language limitations, in any context, and with any yoga variant were considered. Results: The postulated search strings highlighted a total of 69 potentially eligible studies. The study selection system consisted of two levels of screening, (1) abstract selection and (2) full-text selection, for a total of three studies included in the review. The three RCTs included involved mixed treatment sessions in an outpatient setting with a yoga teacher and at home using a DVD. In the various studies, the outcome measures concerned quality of life, ROM, spinal mobility, limb volume, and tissue induration. Conclusions: According to the analysis of the data obtained, yoga as an alternative therapy could be useful if combined with the usual care routine in women with lymphedema related to sensory cancer, in terms of improving physical, professional, and emotional quality of life and reducing symptoms such as fatigue, pain, and insomnia. Furthermore, yoga could bring about a reduction in tissue induration of the limb, greater spinal mobility evaluated in terms of improvement of the pelvic and kyphotic angle, and greater strength in shoulder abduction.

## 1. Introduction

Breast cancer is the most common cancer in women, accounting for 30.0% of all female cancers [1]. Since the late 1990s, there has been a continuous decline in breast cancer mortality rates (−0.8% per year), which can be attributed to the widespread implementation of early detection programs (resulting in earlier diagnoses) and advances in therapeutic approaches [1]. In early invasive breast cancer, neoadjuvant treatments may be recommended before surgery, while adjuvant treatments are advised post-surgery. These treatments can reduce recurrence and mortality rates [2]. Surgical techniques include:

- Conservative surgery: The surgeon removes only the tumor along with a surrounding area of breast tissue (partial resection/quadrantectomy).
- Radical surgery: The surgeon removes the entire breast (mastectomy) [3].

Post-surgical rehabilitation is crucial to:

- Maintain proper arm functionality.
- Prevent muscular and/or skin contractures.
- Avoid improper postures caused by the wound or pain.
- Facilitate the development of collateral lymphatic channels to compensate for the structures removed during surgery [4].

Early mobilization of the upper limb in the weeks following surgery is effective in preventing post-surgical complications such as scar adhesions and lymphedema, as well as issues related to prolonged arm immobilization. Performing specific exercises daily, along with resuming normal daily activities, enables complete recovery of arm mobility. It is advisable to continue mobilization during radiotherapy, which can reduce the elasticity of the irradiated tissues [5]. Despite a gradual decrease in the need for axillary lymph node dissection (ALND) in breast cancer treatment, it remains essential for many patients with lymph node involvement to determine prognosis and guide adjuvant therapies [1]. However, ALND is associated with a significant complication, lymphedema, characterized by fluid accumulation in the soft tissues of the affected arm. Approximately half of the women undergoing ALND experience lymphedema, with some epidemiological estimates exceeding 60% among patients receiving integrated treatments. This has a substantial impact on perceived discomfort, quality of life, body image, social and professional life, and healthcare costs [1]. Lymphedema is marked by edema, or swelling, which can affect one or more limbs (arms in this case), although it is typically unilateral [6,7]. The severity of the edema is classified into stages by the International Society of Lymphology, ranging from stage 0, “sub-clinical” or latent, to stage 3, “irreversible” [6,8,9,10]. Lymphedema often worsens in the summer, before menstruation, or when the limb is kept in a dependent position (standing or sitting) for extended periods [8,11]. Patients report a feeling of heaviness, swelling, skin tightness, burning sensations, discomfort, easy fatigue of the affected limb, and in some cases, itching and pain, with symptoms becoming more pronounced as lymphedema extends [11,12]. Edema can hinder the normal movement of the affected limb, significantly compromising functionality, particularly when a peri-articular region is involved. The emotional and relational impact of lymphedema-related disability is considerable [12,13]. Over time, local skin trophic alterations can occur, including hyperkeratosis (thickening of the epidermis), hyperpigmentation (uniform or patchy skin darkening), increased skin folds, adipose tissue deposits, warts, papillomas, and fungal infections. In severe cases, the edematous limb can become extremely large, resembling a column due to the progressive loss of natural form and definition, with severe hyperkeratosis giving an elephant-like appearance (elephantiasis), a condition more commonly seen in filarial lymphedema [13]. The treatment of lymphedema aims to reduce limb (or region) volume, improve functionality, alleviate symptoms, prevent further lymph accumulation and infections, and enhance the patient’s quality of life [14]. Following treatment, swelling should decrease and symptoms improve, although it may take weeks or months to notice significant improvement. Upon diagnosis, the physiatrist and physiotherapist will explain the multimodal treatment approach for lymphedema: skin care, manual lymphatic drainage, multilayer bandaging, recommended exercises, and the use of elastic compression garments. Some treatments need to be performed daily for best results. Gradually, lymphedema care will become part of the daily routine [12,14]. Lymphedema treatment is based on four fundamental interventions, combined in what is known as comprehensive decongestive therapy and adapted to the affected region:

- Skin care to prevent injuries and infections.
- Manual lymphatic drainage to promote lymphatic flow.
- Compression therapy involving the application of multilayer compression bandages followed by elastic compression garments.
- Therapeutic exercise including specific muscle exercises to improve lymphatic drainage and joint mobility, as well as respiratory exercises to enhance muscle strength [14].

Breast cancer patients with secondary lymphedema commonly use complementary and integrative therapies for support during cancer treatment and to manage treatment-related side effects. Over the years, positive results have been observed in terms of anxiety/stress, depression/mood disorders, fatigue, quality of life/physical functioning, chemotherapy-induced nausea and vomiting, lymphedema, chemotherapy-induced peripheral neuropathy, pain, and sleep disorders through the use of integrative therapies such as music therapy, meditation, stress management, yoga, acupressure, and acupuncture [15,16,17]. Traditional yoga is a holistic practice that aligns the body and mind. Modern yoga typically comprises three elements in various combinations: physical postures (e.g., warrior pose), breathing techniques (e.g., alternate nostril breathing), and meditation/mental relaxation (e.g., chakra meditation). In recent years, yoga has garnered global attention from both the scientific community and the general population due to its numerous health benefits [18]. In the past decade, there has been an increase in studies on the effects of yoga on women who have undergone breast cancer surgery and developed lymphedema. We aim to provide a comprehensive review of yoga’s benefits for managing lymphedema in breast cancer survivors. While yoga offers significant improvements in tissue induration, spinal mobility, and shoulder strength, it is essential to recognize other emerging treatments for lymphedema. Surgical interventions [19,20,21,22], such as lymphatico-venicular anastomosis and vascularized lymph node transfers, have shown promising results. Additionally, pharmacotherapy, including anti-inflammatory drugs and cell-based therapies, offers new avenues for treatment [23]. Incorporating these treatments into a holistic approach can enhance patient outcomes and provide a more comprehensive management strategy. A literature review identified four systematic reviews, published between 2019 and 2023, evaluating the effects of yoga on women with breast cancer-related lymphedema. The most recent review, published in 2023 on Web of Science, included studies up to 2021, and all reviews included RCTs and studies of low methodological quality.

## 2. Methods

The present scoping review was conducted following the JBI methodology [24] for scoping reviews. The Preferred Reporting Items for Systematic reviews and Meta-Analyses extension for Scoping Reviews (PRISMA-ScR) [25] Checklist for reporting was used.

### 2.1. Review Question

We formulated the following research question: “What are the effects of yoga on women with breast cancer-related lymphedema as reported in randomized controlled trials (RCTs)”.

### 2.2. Eligibility Criteria

Studies were eligible for inclusion if they met the following Population, Concept, and Context (PCC) criteria.

Population (P): Women with breast cancer who have developed secondary lymphedema.

Concept (C): Any form of yoga intervention.

Context (C): Studies conducted in any setting.

### 2.3. Exclusion Criteria

Studies that did not meet the specific PCC criteria were excluded.

### 2.4. Search Strategy

An initial limited search of MEDLINE via PubMed identified relevant articles and their index terms. These terms were then used to create a comprehensive search strategy for MEDLINE, which was adapted for Cochrane Central, Scopus, and PEDro. Additionally, the grey literature and reference lists of relevant studies were searched. All searches were conducted on 30 June 2024, with no date limitations.

PubMed: “breast cancer” AND “lymphedema” AND “yoga” with MeSH terms, filters: RCT, clinical trial.

Scopus: (“breast cancer” OR “breast carcinoma” OR “mammary cancer”) AND (“lymphedema” OR “lymphoedema”) AND (“yoga”), filter: article.

Cochrane: lymphedema yoga.

Web of Science: (“breast cancer” OR “breast carcinoma” OR “mammary cancer”) AND (“lymphedema” OR “lymphoedema”) AND (“yoga”), filter: article.

Pedro: (“breast cancer” OR “breast carcinoma” OR “mammary cancer”) AND (“lymphedema” OR “lymphoedema”) AND (“yoga”), filter: article.

### 2.5. Study Selection

The process used a systematic approach for selecting studies for a scoping review. Initially, search results were collected and refined with Zotero, removing duplicates. Screening involved two levels: title and abstract review, followed by assessment of the full text, both conducted independently by two authors with a third resolving discrepancies. The selection followed PRISMA 2020 guidelines to ensure transparency and reliability. This method aimed to identify articles relevant to the research question.

### 2.6. Data Extraction and Data Synthesis

Data for the scoping review were extracted using a JBI-based form, capturing key details such as authorship, publication country and year, study design, patient characteristics, outcomes, interventions, and procedures. Descriptive analyses were conducted, presenting the study distribution numerically. The review process was summarized in tables to enable easy comparison and understanding of the studies’ main aspects and findings.

## 3. Results

**Figure 1:**
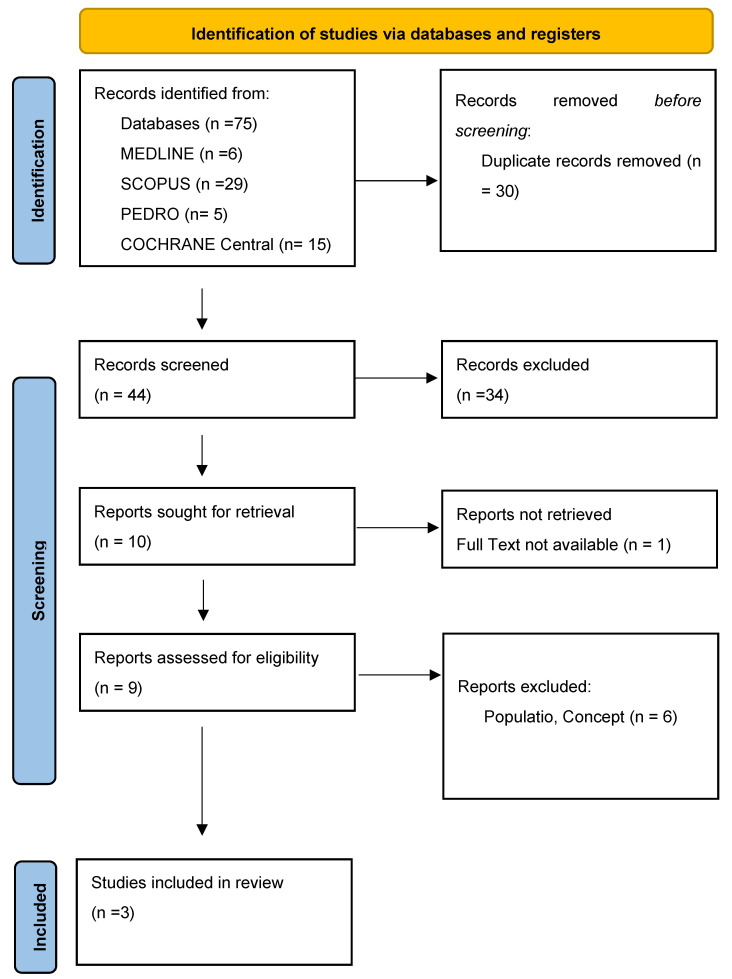
Preferred reporting items for systematic reviews and meta-analyses 2020 (PRISMA) flow diagram.

As presented in the PRISMA 2020 flow diagram (Figure 1), from 75 records identified by the initial literature searches, 72 were excluded and three articles were included (Table 1, Table 2 and Table 3). The quality of the studies was assessed with the PEDro scale (Table 4) and ROB2 (Table 4).

In these three RCTs, yoga sessions were conducted both with an instructor and at home via DVD, compared to routine care alone. In the study by Nilofar Pasyara et al. (2019) [26], the yoga exercise protocol included 20 yoga asanas and five breathing exercises. The authors selected these exercises for their role in expanding the chest, maximizing the range of motion of the neck, shoulders, and elbows, and stretching the skin by activating muscles around the armpits and lymph nodes. Participants were also provided with a booklet on the benefits of yoga exercises for body structure. In the studies by Annette Loudon et al. (2016) [27] and Annette Loudon et al. (2014) [28], the Satyananda Yoga style was chosen for its systematic and progressive practices based on an integrated system of pranayama, asana, meditation, and relaxation, with possible modifications. The practices were selected following the principles of manual lymphatic drainage, with gentle actions on the ROM of the shoulder and spine, focusing on posture and movement patterns involving core and shoulder stabilizers, and reducing stress [27,28]. In all three studies, yoga sessions were conducted at least once a week with the instructor and at home via DVD on the remaining days. The setting could be outpatient or home-based. The duration of the yoga program varied from 90 min with the instructor and 45 min with the DVD [27,28] to the time needed for the individual to complete the exercise protocol [26]. Comparisons in the three RCTs included routine care alone.

### 3.1. Outcomes and Outcome Measures

The outcome measures used in the included studies were: EORTC QLQ-C30 [26], water displacement [26], two-arm goniometer [27], dynamometer [27], video analysis [27], circumferential measurement, BIS L-dex (bioimpedance spectroscopy), digital tonometer, VAS (Visual Analog Scale), and LYMQOL [28].

The EORTC Core Quality of Life (EORTC QLQ-C30) questionnaire is designed to measure the physical, psychological, and social functions of cancer patients. It comprises multi-item scales and single items [29]. This questionnaire evaluates the life quality of cancer patients and includes nine multi-item scales: five functional scales (physical, role, cognitive, emotional, and social), three symptom scales (fatigue, pain, and nausea and vomiting), and a global health status scale. Additionally, there are six single items concerning dyspnea, insomnia, appetite loss, constipation, diarrhea, and financial difficulties. Each scale is scored from 0 to 100. Higher scores on functional scales indicate higher levels of functioning, while higher scores on symptom scales indicate greater symptomatology [26]. The VAS (Visual Analog Scale) is a visual analog scale that measures pain intensity. It consists of a 10 cm line with two endpoints representing 0 (“no pain”) and 10 (“worst pain imaginable”) [30,31]. Water displacement involves immersing the lymphedematous limb in a graduated tank of water to measure the volume accurately based on the displaced liquid. The dynamometer is an instrument used to measure the ability to sustain physical effort over time and its intensity. The tonometer is used as a precise and repeatable method for assessing tissue tone by measuring tissue resistance to compression and its variation over time. The instrument consists of a base, a metal cylinder, and a 200 g weight that induces the penetration of a pin into the tissue, with two measurement dials. The tonometer is placed on the skin surface of the limb, held vertically without applying pressure, and readings are taken at constant marker points after 5 s [32]. BIS (Bioimpedance spectroscopy) is a non-invasive, low-cost technology that accurately measures intra- and extracellular fluid volumes in a clinical setting. The L-Dex score represents the difference in extracellular fluid between an at-risk limb and a non-affected limb [33]. The LYMQOL (Lymphedema Quality of Life Questionnaire) assesses the quality of life of individuals with upper limb lymphedema due to breast cancer. The self-administered questionnaire includes 21 questions evaluating the impact of lymphedema on quality of life across four domains: activity, physical, symptoms, and emotional aspects. Each question has four response options, with higher scores indicating poorer quality of life. The total score for each domain is calculated by summing all scores in that domain and dividing by the total number of responses [34]. In study [26], the intervention group received a yoga program combined with routine lymphedema care, while the control group received only routine care. In study [27], the intervention group received in-person yoga sessions and home yoga via DVD in addition to routine care, whereas the control group received only routine care. In study [28], the comparison was between patients receiving yoga exercises plus routine care and those receiving routine care alone.

### 3.2. Synthesis of Results

Due to the limited number of studies and evaluated outcomes, a qualitative analysis of the results is presented narratively and, where possible, in tabular/graphical form.

### 3.3. Quality of Life

Nilofar Pasyara et al. [26] reported the results for the EORTC QLQ-30 and water displacement measures. A total of 40 women with breast cancer-related lymphedema participated in the study, with 20 in the intervention group and 20 in the control group. Outcomes were measured at baseline, 4 weeks, and 8 weeks post-intervention. At 4 weeks, a significant difference was observed in the “role functioning subscale” between the groups, with a *p*-value of 0.03. At 8 weeks, there was a significant difference in physical and emotional function, as well as in fatigue, pain, insomnia, and financial difficulties. No significant differences were found in arm volume at either 4 or 8 weeks.

Annette Loudon et al. (2016) [27] and Annette Loudon et al. (2014) [28] conducted a multicenter RCT to evaluate the effects of yoga on quality of life, ROM, and strength. The studies enrolled 28 participants, randomized into intervention (n = 15) and control (n = 13) groups. Measurements were taken at baseline, 8 weeks, and 12 weeks (4 weeks post-intervention). This was measured using the LYMQOL questionnaire, which showed notable enhancements in both the physical and emotional well-being of the participants.

### 3.4. Tissue Induration

In the 2014 study by Annette Loudon et al. [28], the primary outcome was arm volume, measured in terms of circumference and extracellular fluid (bioimpedance spectroscopy). Secondary outcomes included tissue consistency, sensation level, pain, fatigue, limiting effects, and quality of life (LYMQOL). Results showed a significant decrease in tissue induration (3.20 points) in the intervention group compared to the control group, with a significant *p*-value (*p* = 0.050). There was also a significant reduction in the LYMQOL symptom subscale (*p* = 0.038). No significant differences in arm volume were found between the groups at 8 weeks, with an increase in arm volume in the intervention group compared to the control group (*p* = 0.032).

### 3.5. Range of Motion and Strength

In the 2016 study by Annette Loudon et al. [27], ROM, strength, and spinal mobility were assessed using a goniometer, dynamometer, and video analysis. Significant findings include:

- Shoulder abduction ROM for the non-affected limb at 8 weeks showed a mean difference (MD) of −14.70° (95% CI: −29.30° to −0.10; *p* = 0.049).
- Shoulder flexion ROM for the non-affected limb at 8 weeks had an MD of −19.00° (95% CI: −33.65° to −4.36; *p* = 0.011).
- Internal rotation of the affected limb decreased from weeks 8 to 12 in the intervention group with an MD of −10.97° (95% CI: −17.37 to −4.56; *p* = 0.001).
- Shoulder abduction strength increased in the intervention group at 8 weeks for the affected limb (MD 9.5 kg; 95% CI: 0.34 to 18.66; *p* = 0.042) and the non-affected limb (MD 11.58 kg; 95% CI: 0.25 to 22.91; *p* = 0.045).
- Pectoralis major strength in the non-affected limb decreased from weeks 8 to 12 in the intervention group (MD −11.80 N; 95% CI: −19.21 to −4.38; *p* = 0.002).
- Serratus anterior strength increased significantly at 8 weeks, while pectoralis minor strength decreased significantly at 12 weeks.
- Handgrip strength showed no significant differences at baseline and 8 weeks but decreased in the affected limb at 12 weeks (MD 3.58 kg; 95% CI: 1.50 to 5.67; *p* = 0.01).

### 3.6. Spinal Mobility

At baseline, the intervention group had a higher pelvic angle than the control group (MD +9.97°; 95% CI: 2.76 to 17.17; *p* = 0.007). By 8 weeks, the intervention group showed an improvement with a decreased pelvic angle compared to the control group (MD −8.39°; 95% CI: −15.64 to −1.13; *p* = 0.023), primarily due to reduced pelvic obliquity (MD −9.96°; 95% CI: −14.54 to −5.37; *p* = 0.001). The thoracic kyphosis angle was initially higher in the intervention group (MD 8.13°; 95% CI: −0.10 to 16.37; *p* = 0.053), and by 8 weeks, there was a trend towards improvement (MD −3.88°; 95% CI −8.08 to 0.32; *p* = 0.070). No significant differences in spinal mobility were noted between the groups at 12 weeks [27].

**Table 4:** Quality Assessment using PEDro and RoB-2 Scales.

Author and Year	PEDro Score (Out of 11)	Random Sequence Generation	Allocation Concealment	Blinding of Participants and Personnel	Missing Outcome Data	Measurement of the Outcome	Reported Result	Overall Risk of Bias
Nilofar Pasyara et al., 2019 [26]	10	Low risk	Low risk	Some concerns	Low risk	Low risk	Low risk	Unclear risk
Annette Loudon et al., 2016 [27]	10	Low risk	Low risk	Some concerns	Low risk	Low risk	Low risk	Unclear risk
Annette Loudon et al., 2014 [28]	10	Low risk	Low risk	Some concerns	Low risk	Low risk	Low risk	Unclear risk

This table summarizes the quality assessment of the included randomized controlled trials (RCTs) using the PEDro and RoB-2 scales, detailing the methodological rigor and risk of bias. Legend: PEDro Score: Physiotherapy Evidence Database Score, RoB-2: Risk of Bias 2 Tool.

## 4. Discussion

This review aimed to evaluate the effects of yoga on women with breast cancer-related lymphedema, synthesizing findings from three randomized controlled trials (RCTs) and several systematic reviews. The variability in outcomes across the included studies necessitated a qualitative analysis. Despite methodological differences, the studies collectively highlight the potential benefits of yoga in this patient population. Nilofar Pasyara et al. [26] demonstrated that yoga significantly improved various aspects of quality of life, including physical and emotional functioning, and reduced symptoms such as fatigue, pain, and insomnia. These findings underscore the holistic benefits of yoga, which align with its emphasis on physical postures [35], breathing techniques [36], and mental relaxation [37]. However, no significant changes in arm volume were observed, suggesting that while yoga may enhance overall well-being, its impact on lymphedema-related swelling may be limited. Yoga offers several advantages over conventional massage. Unlike massage, which primarily focuses on manipulating soft tissues to improve circulation and reduce muscle tension, yoga integrates physical postures, breathing exercises, and meditation. This holistic approach not only aids in physical rehabilitation by improving flexibility, strength, and lymphatic flow but also enhances mental well-being by reducing stress and promoting relaxation. The integration of breath control and meditation in yoga can lead to better overall outcomes in managing lymphedema, such as reducing fatigue and pain, which are not directly addressed by massage therapy [38].

The combination of yoga with conventional surgical therapy or pharmacotherapy could potentially offer comprehensive benefits. Post-surgical yoga can aid in quicker recovery by improving mobility and reducing scar tissue formation. When combined with pharmacotherapy, yoga can help in managing side effects such as fatigue, pain, and insomnia, thereby improving the overall quality of life. This integrated approach may enhance the effectiveness of traditional treatments and provide a more balanced and holistic recovery process for patients.

In contrast, the studies by Annette Loudon et al. [27,28] focused more on physiological outcomes. The 2014 study [28] reported significant reductions in tissue induration and improvements in the symptom subscale of the LYMQOL questionnaire, indicating that yoga can positively affect tissue characteristics and quality of life. Interestingly, no significant differences in arm volume were found, and an unexpected increase in volume was noted in the intervention group, suggesting that while yoga may improve certain aspects of lymphedema, it might not consistently reduce limb swelling.

The 2016 study [27] further expanded on these findings by examining range of motion (ROM), strength, and spinal mobility. The results indicated that yoga improved shoulder strength and spinal mobility, particularly in reducing pelvic and thoracic angles, which may contribute to better posture and reduced physical strain. These improvements in musculoskeletal function are critical, as they can enhance daily functioning and reduce discomfort associated with lymphedema.

The systematic reviews corroborated these findings, highlighting the overall positive effects of yoga on quality of life, ROM, and musculoskeletal symptoms. Wei et al. (2019) [39] noted improvements in lymphedema and spinal mobility, although inconsistencies across studies prevented a definitive conclusion on the superiority of yoga over routine care. Wanchai et al. (2020) [40] suggested that not all yoga types are equally effective, pointing to the need for more rigorous research. Saraswathi et al. (2021) [41] and Levenhagen et al. (2023) [42] supported the safety and acceptability of yoga, with no reports of exacerbated lymphedema, reinforcing its potential as a complementary therapy. Despite these promising findings, the review also highlights significant limitations. The limited number of RCTs and their small sample sizes constrain the generalizability of the results. The uncertain risk of bias, as evaluated by the RoB-2 tool, further complicates the interpretation of these findings. Moreover, the absence of published results from completed RCT protocols in the grey literature suggests potential publication bias or other issues affecting data availability.

In conclusion, while yoga shows promise in improving quality of life, musculoskeletal function [43], and some aspects of lymphedema management in women with breast cancer-related lymphedema, further high-quality, large-scale studies are needed to substantiate these findings and clarify its role in reducing limb swelling. The integration of yoga into therapeutic plans for breast cancer-related lymphedema appears to be a viable and beneficial approach, warranting continued research and clinical application.

### Clinical Practice Implications

The integration of yoga into the management of breast cancer-related lymphedema shows promise for improving patient outcomes. Yoga has been found to enhance quality of life, alleviate symptoms such as fatigue, pain, and insomnia, and improve musculoskeletal function. Personalized yoga programs, involving both in-person and at-home sessions, can be tailored to meet individual patient needs. Educating patients about the benefits of yoga and providing ongoing support can enhance adherence and effectiveness. Collaboration between healthcare providers and certified yoga instructors is crucial for safe implementation, and continuous monitoring of patient progress is essential. Further research with larger sample sizes and standardized protocols will help establish the long-term benefits of yoga in this patient population.

## 5. Conclusions

The integration of yoga into the management of breast cancer-related lymphedema can enhance quality of life, reduce symptoms such as fatigue and pain, and improve musculoskeletal function. Personalized yoga programs, patient education, and interdisciplinary collaboration are essential for effective implementation. Further research is needed to confirm its long-term benefits and efficacy in reducing limb swelling.

## Figures and Tables

**Table 1:** Main characteristics of included studies.

Author and Year	Study Design	Language of Publication	Baseline Population	Intervention Group	Control Group	Outcome	Measure
Nilofar Pasyara et al., 2019 [26]	RCT	English	All women diagnosed with breast cancer-related lymphedema	Yoga program and routine lymphedema care	Routine care only (lymphatic drainage, compression garments)	Quality of life	EORTC QLQ-C30
						Arm volume	Water displacement method
Annette Loudon et al., 2016 [27]	RCT	English	Upper limb lymphedema related to stage 1 breast cancer (International Society of Lymphology)	In-person yoga program, home yoga DVD, and routine care	Routine care only (lymphatic drainage, compression garments)	Shoulder ROM	Two-arm goniometer
						Strength	Dynamometer
						Spinal mobility	Video analysis
Annette Loudon et al., 2014 [28]	RCT	English	Upper limb lymphedema related to stage 1 breast cancer (International Society of Lymphology)	In-person yoga program, home yoga DVD, and routine care	Routine care only (lymphatic drainage, compression garments)	Arm volume	Non-elastic Jobst tape + cone formula
						Extracellular fluids	Bioimpedance spectroscopy
						Tissue induration	Digital tonometer
						Pain and fatigue	Visual Analog Scale (VAS)
						Quality of life	LYMQOL

This table summarizes the characteristics of the randomized controlled trials (RCTs) included in the review, highlighting the study design, language of publication, baseline population, intervention and control groups, outcomes, and outcome measures. Legend: EORTC QLQ-C30: European Organisation for Research and Treatment of Cancer Quality of Life Questionnaire-C30, LYMQOL: Lymphedema Quality of Life Questionnaire, RCT: Randomized Controlled Trial, ROM: Range of Motion, VAS: Visual Analog Scale.

**Table 2:** Baseline Population Characteristics.

Author and Year.	Age (Years, Mean)	Diagnosis	Lymphedema Stage	Total Number of Participants
Nilofar Pasyara et al., 2019 [26]	51.6	Breast cancer-related lymphedema	Stages 1, 2, and 3	40
Annette Loudon et al., 2016 [27]	57.6	Breast cancer-related lymphedema	Stage 1	28
Annette Loudon et al., 2014 [28]	57.6	Breast cancer-related lymphedema	Stage 1	28

This table presents the baseline characteristics of the populations in the included studies, detailing the mean age, diagnosis, stage of lymphedema, and total number of participants.

**Table 3:** Intervention Characteristics.

Author and Year	Intervention	Comparison	Setting	Frequency and Duration	Intensity and Volume	Training Period
Nilofar Pasyara et al., 2019 [26]	Yoga exercise protocol and routine care	Routine care only	2 sessions with a yoga instructor, 1 at home via DVD	3 sessions per week for 8 weeks	-	At least one year after breast surgery
Annette Loudon et al., 2016 [27]	Yoga exercises and routine care	Routine care only	Both outpatient and home settings	1 session per week with instructor and at home via DVD for 8 weeks	Weekly session with instructor (90 min), home sessions (45 min)	At least 6 months after completing breast cancer treatment
Annette Loudon et al., 2014 [28]	Yoga exercises and routine care	Routine care only	Both outpatient and home settings	1 session per week with instructor and at home via DVD for 8 weeks	Weekly session with instructor (90 min), home sessions (45 min)	At least 6 months after completing breast cancer treatment

This table outlines the characteristics of the yoga interventions in the included studies, detailing the intervention and comparison groups, setting, frequency and duration, intensity and volume, and the training period. Legend: DVD: Digital Versatile Disc.
